# Weaker neuroligin 2–neurexin β1 interaction tethers membranes and recruits gephyrin at membrane junctions through clustering

**DOI:** 10.1126/sciadv.ads9732

**Published:** 2026-03-13

**Authors:** Robbie Boyd, Khuloud Jaqaman, Weiwei Wang

**Affiliations:** ^1^ Department of Biophysics, University of Texas Southwestern Medical Center, Dallas, TX, USA.; ^2^ Lyda Hill Department of Bioinformatics, University of Texas Southwestern Medical Center, Dallas, TX, USA.

## Abstract

Single-pass transmembrane proteins neuroligin (NL) and neurexin (NRX) constitute a pair of synaptic adhesion molecules that are essential for the formation of functional synapses. Binding affinities vary by ~1000-fold between combinations of NL and NRX subtypes, which contribute to chemical and spatial specificities. Among major NL-NRX subtypes, NL2 and NRXβ1 have the lowest affinity. Here, we report structures of NL2 in complex with NRXβ1 in several conformations, along with NL2 alone. We identify mechanisms underlying the modulation of NL-NRX affinities and how the weaker NL2-NRXβ1 interaction alone is capable of tethering lipid membranes. We further show that NL2 and NRXβ1 cluster at intercellular junctions and recruit the master postsynaptic scaffolding protein gephyrin, which further clusters neurotransmitter receptors. These findings suggest a dual role of the NL2-NRXβ1 interaction—both as mechanical tether and as signaling receptor—to ensure correct spatial and chemical coordination between two cells to generate functional synapses.

## Teaser

Despite low affinities to neurexin due to structural features, neuroligin 2 promotes synapse formation through avidity effect.

## INTRODUCTION

Neuronal cells transmit and process information through synapses, where the plasma membranes of the pre- and the postsynaptic neurons are tethered by synaptic adhesion molecules (*1*, *2*). Postsynaptic protein neuroligin (NL) and presynaptic protein neurexin (NRX) constitute a pair of synaptic adhesion molecules that play indispensable roles in synapse function (*1*, *3*, *4*). Knockout of NL or NRX results in defects in synaptic transmission, with triple knockout of NL family proteins being fatal for postnatal mice (*5*–*8*). Genetic variants in humans are associated with neuropsychiatric disorders including autism, intellectual disabilities, and epilepsy (*9*–*14*). Although not seeming to change the number of synapse-like structures in some tissues, genetic ablation of NL and NRX reduces synapse function throughout the nervous system (*2*, *15*–*17*).

Multiple NL and NRX paralogs are found in mammalian nervous systems. NL has four major paralogs, NL1 to N4. NL1 and NL2 preferentially localize in excitatory and inhibitory synapses, respectively, while NL3 (*18*, *19*) and NL4 (*20*, *21*) show less/unclear selectivity. The NRX family contains three paralogs, NRX1 to NRX3; each have a long “α” form and a short “β” form that originate from distinct promoters and thus alternative transcriptional start sites. The “α” form contains six extracellular “laminin, neurexin and sex hormone – binding globulin–like” (LNS) domains, and the “β” form contains only the last LNS domain that binds with NL. Combined with alternative splicing, more than a thousand splice variants are possible for NRX, and these differ in affinity for NL and other ligands. NL1 and NL4 bind to NRX with nanomolar to submicromolar affinities, while NL2 and NL3 bind with weaker affinity in the micromolar range (*22*, *23*). The wide range of NL-NRX interaction affinities have been implicated in the chemical specificity of synapses (*8*, *24**–**26*).

A few fundamental questions remain unanswered regarding how the NL-NRX interaction works at synapses. NL and NRX are both single-pass transmembrane proteins. Structural information to date is limited to truncated (or possibly degraded) NL and NRX containing only the extracellular domains (ECDs) for NL and the LNS domain 6 for NRX (*10*, *23*, *27**–**33*). Although higher-affinity structures of NL1 and NL4 in complex with NRX have been resolved almost 20 years ago (*27*, *28*), lower-affinity structures of NL2 and NL3 in complex with NRX have not been reported to date. Because of the lack of structural data, the mechanism underlying the lower-affinity interaction has been obscured. This has put into question whether these interactions allow for a stable complex and whether they are sufficient to tether cellular membranes.

To address the above questions, we resolved structures of NL2 alone and in complex with NRXβ1. The constructs used do not contain a splice insert at site A in NL2 (*34*, *35*) and the splice site 4 (*36*, *37*) in NRXβ1 since they disrupt interaction (please see Materials and Methods for details). These structures suggest flexible orientations between ECDs and transmembrane domains (TMs) for both NL and NRX, as well as within dimeric NL2 ECD. Examination of the structures show that the mechanism underlying low NL2-NRXβ1 binding affinity differs from that which was previously proposed (*29*). We further show that NL2 and NRXβ1 are clustered at intercellular junctions in non-neuronal cells and that their interaction alone is capable of tethering two lipid membranes; this suggests a signaling role leading to the formation of synapses. In addition, we show coclustering of NL2, gephyrin, and glycine receptor at the cellular membrane, which suggests a role in recruiting neurotransmitter receptors to the synapse and in signaling presynaptic cell through clustering of NRX. On the basis of these results, we propose a mechanism through which the weaker NL2-NRXβ1 interaction both tethers membranes and signals the recruitment of other synaptic components through clustering.

## RESULTS

### Flexible linker between ECD and TM

**Fig. 1.**
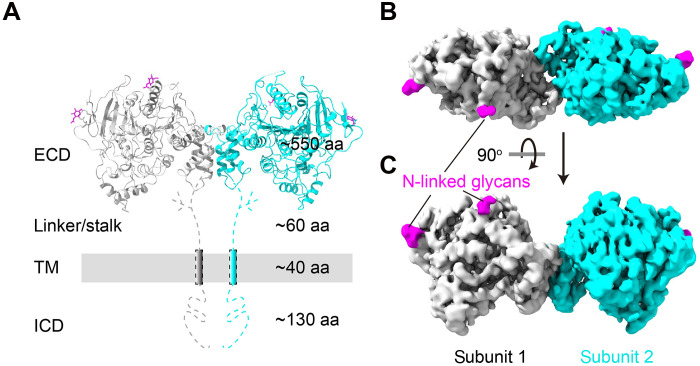
Structure of full-length NL2 contained only the ECD. (**A**) Schematic of full-length NL2. Gray bar denotes postsynaptic membrane. Cryo-EM density map of NL2 viewed (**B**) down extracellular side and (**C**) parallel to membrane. Two subunits are colored in gray and cyan, respectively. N-glycans are colored in purple. aa, amino acids.

In full-length NLs, the Ser- and Thr-rich linker (~60 amino acids) between the ECD (~550 amino acids) and the transmembrane helix (~40 amino acids; TM) is named the stalk domain; it contains O-glycosylation sites and may provide some structural rigidity and spacing between the ECD and TM (*23*, *29*, *31*, *38*) (Fig. 1A). The intracellular domain (ICD; ~130 amino acids) that interacts with multiple synaptic proteins is believed to be unstructured. Reported structures of NLs were determined using truncated proteins containing only the ECD (*10*, *27**–**33*). We analyzed full-length NL2 using single-particle cryo–electron microscopy (cryo-EM) to examine whether the ECD has defined/preferred orientations with respect to the stalk/TM/ICD (*23*).

EM densities for the stalk, TM (detergent micelle) and ICDs are absent for full-length NL2 showing no degradation (figs. S1, A, B, and E, and S2, A and B). Only the ECD is resolved, at an overall resolution of 3.3 Å (Fig. 1B and fig. S2, C to F). The density map quality allows for unambiguous model building across different regions of the ECD (Fig. 1A; see Materials and Methods for details). The map shows that NL2 forms a dimer through the ECD, like that observed in ECD-only structures (*10*, *11*, *27*, *28*, *32*, *33*, *39*). Apparently, the stalk domain provides high flexibility between ECD and TM. This is consistent with previous findings, where x-ray and neutron scattering methods also pointed to the flexible nature of the stalk domain (*23*). This observation adds NL and NRX proteins to the list of single-pass transmembrane receptors, including receptor tyrosine kinases, T cell receptors, and many others, that show flexible ECD-TM orientations (*40*, *41*).

### Full- and substoichiometric complexes of NL2-NRXβ1 with structural flexibility

Among NL subtypes NL1 to NL4, NL2 has the lowest apparent affinity with NRXβ1 (*22*, *23*). Structures of NL1-NRXβ1 [dissociation constant (*K*_d_) ~ 10 nm] and NL4-NRXβ1 (*K*_d_ ~ 100 nm) ECD complexes have been resolved previously (*23*, *27*, *28*). NL3 binds NRXβ1 with ~1 μM *K*_d_, and NL2 binds even weaker at ~10 μM *K*_d_ (*22*, *23*). Thus far, no NL2/NL3-NRX complex structure has been reported. Whether the weaker NL2/3-NRXβ1 interaction enables a stable complex has not been established.

**Fig. 2.**
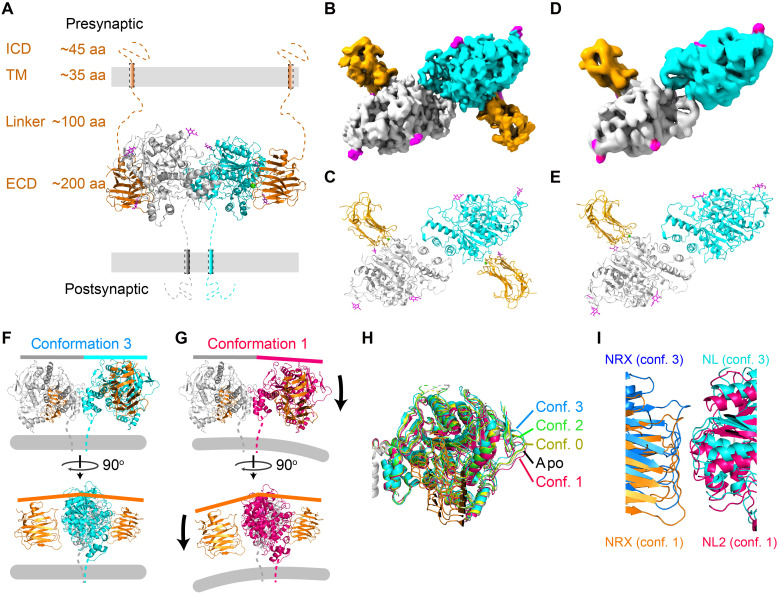
Overall conformations of full- and substoichiometric NL2-NRXβ1 complexes. (**A**) Illustration of NL2-NRXβ1 complex at synapse. Two NL2 subunits are colored in gray and cyan, respectively. NRXβ1 and N-glycans are colored in orange and purple, respectively. (**B**) Density map and (**C**) atomic model of a 2:1 NRXβ1:NL2 dimer full stoichiometric complex. (**D**) Density map and (**E**) atomic model of the 1:1 NRXβ1:NL2 dimer substoichiometric complex. Different orientations between two NL2 subunits in (**F**) conf. 3 and in (**G**) conf. 1. Gray bars indicate direction of postsynaptic membrane. (**H**) Comparison of one NL2 subunit orientation across all conformations, with the other subunit aligned (gray, aligned subunit). Color codes shown. (**I**) NRXβ1 and NL2 move together and preserve interaction interface.

Mixing of purified full-length NL2 and NRXβ1 led to a coelution peak (peak 2) and an additional peak (peak 3) mostly composed of NRXβ1 in size exclusion chromatography (fig. S1, B to D). Similar to NL2 alone, single-particle cryo-EM analysis resolved the complex of NRXβ1 and NL2 ECDs only (fig. S2, G to M), suggesting a flexible connection between the ECDs and TMs for both proteins (Fig. 2A). Three conformations of a full stoichiometric (2:1 NRXβ1:NL2 dimer) complex, differing in the relative orientations between the two subunits of the NL2 dimer, were resolved at a 3.2-Å overall resolution (Fig. 2, B and C, and fig. S2, K to M, conf. 1 to 3). A separate substoichiometric complex (1:1 NRXβ1:NL2 dimer) was also resolved at a 3.9-Å overall resolution (Fig. 2, D and E, and fig. S2, K to M, conf. 0).

The NL2 dimer shows structural flexibility between its two monomers. When comparing the three structures of full stoichiometric complexes (fig. S2M, conf. 1 to 3), a pivoting motion between the two monomers becomes evident. This motion leads to a change in orientation between the two monomers when viewed in parallel to the membrane (Fig. 2, F and G). The substoichiometric complex (conf. 0) has a very similar orientation as apo NL2, both sitting roughly in the center of the range observed across the full stoichiometric complexes (Fig. 2H). NL2 and NRXβ1 move in coordination across all the complex structures and maintain an unchanged interaction interface (Fig. 2I). Since the binding of NRXβ1 does not result in a unidirectional change in the NL2 monomer orientation, the pivoting motion is likely arising from intrinsic NL2 flexibility. A similar pivoting motion has been reported between the NL2 and NL3 ECDs (*10*), suggesting that this may be a universal feature in the NL family of proteins. Heparan sulfate (HS) tethered to full-length NRXβ1 has been proposed to bind near the NL2 ECD dimer interface (*42*). However, EM density is not observed near this site, which may result from flexible nature of interaction or the absence of HS binding. In the case that HS binds at this site, changes in NL2 monomer orientations could be related.

The flexibility between the two NL2 homomers is indicated from published NL2 structures. Rodent NL2 dimers (*32*, *43*) have been shown to have orientations that fall within the range that we observed across conf. 1 and conf. 3 and independent of whether x-ray crystallography or cryo-EM was used (fig. S4A). Human NL2 (*10*, *33*) may have slightly more flexibility since it (*10*) sits outside of the range defined by conf. 1 and conf. 3.

### Mechanisms underlying weak NL2-NRXβ1 interaction

**Fig. 3.**
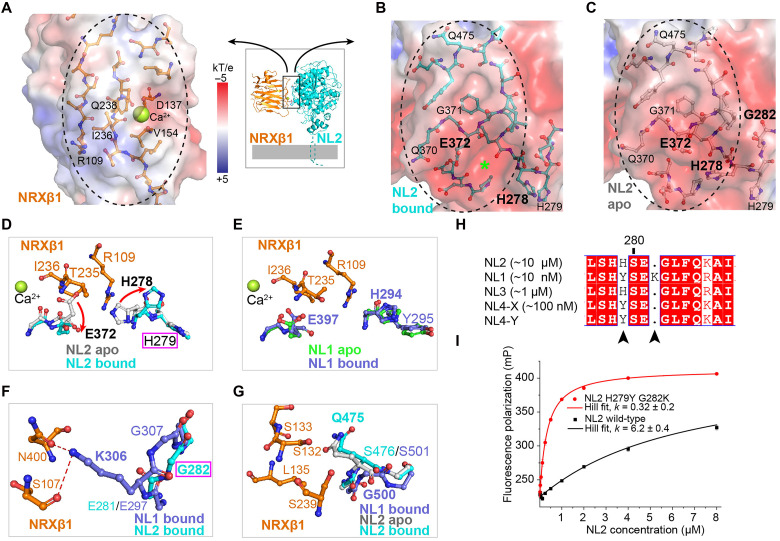
Structural features contributing to lower NL2-NRXβ1 affinity. Binding interfaces of (**A**) NRXβ1, (**B**) NL2 bound, and (**C**) NL2 apo. Molecular surfaces are colored according to electrostatics calculated using Adaptive Poisson-Boltzmann Solver (APBS) plugin in PyMOL (*87*). Amino acid residues at the interfaces are shown as sticks. Green star indicates cavity in bound NL2 that interact with NRXβ1:R109, which is buried in apo NL2. (**D**) Conformational changes of E372 and H278 between bound and apo NL2 open or occlude NRXβ1:R109 binding cavity. (**E**) In NL1, the NRXβ1:R109 binding cavity is present in both apo [Protein Data Bank (PDB) ID: 3BIX] and bound (PDB ID: 3BIW) states. (**F**) NL1:K306 makes polar contacts with NRXβ1 but is substituted to G282 in NL2. (**G**) NL1:G500 substitution to NL2:Q475 does obstruct NRXβ1 binding or change conformation from apo to bound states. (**H**) Sequence alignment of human neuroligins [B (−) variant whenever applicable], with reported NRXβ1 *K*_d_ ranges shown. S280 is indicated for NL2, and two substitutions are highlighted by black arrows. (**I**) Fluorescence polarization of NRXβ1 upon binding with wild-type NL2 (black) or H279Y G282K mutant (red), fitted with Hill equation with *k* values indicated and shared slope *n* = 1.05 ± 0.05.

The NL2-NRXβ1 interaction interface resembles that of NL1-NRXβ1, composed of mostly polar interactions with a buried-area of ~1000 Å^2^ (1160 Å^2^ for NL1-NRXβ1) and with a Ca^2+^ mediating multiple interactions (Fig. 3, A and B). NRXβ1 uses an essentially identical binding site to form NL1 and NL2 complexes. Ca^2+^-mediated interactions are also strictly conserved (*27*, *44*). However, two unique features of NL2 likely contribute to its much lower affinity.

First, the interaction with NRXβ1 requires binding site rearrangements in NL2 but not NL1 (Fig. 3, B to D). R109 of NRXβ1 binds to a negatively charged cavity on NL2 between E372 and H278 (Fig. 3B, green star). However, in apo state NL2, E372 and H278 side chains occupy this cavity, preventing NRXβ1:R109 from binding (Fig. 3D). In contrast, this cavity is available and does not change between the apo and bound states of NL1 (*27*, *29*) (Fig. 3E). The change of NL2 H278 conformation, which moves away when NRX binds, seems only related to NRX binding; NL2 alone (*10*, *27*) or in complex with MAM domain-containing glycosylphosphatidylinositol anchor (MDGA) (*32*, *33*) has H278 obstructing NRX binding pocket independent of species (fig. S4B).

In analyzing why apo state NL2:H278 adopts a different conformation than the corresponding NL1:H294 (Fig. 3, D and E), we found that the adjacent residue is not conserved, namely, H279 in NL2 and Y295 in NL1. We reason that in NL2, H278 and H279 will carry the same charge and cause repulsion, while in NL1, H294 and Y295 will not repel each other, leading to the difference in NL2:H278/NL1:H294 conformations in the apo state (see fig. S4, E and F).

Second, a nonconserved residue, NL2:G282 compared with NL1:K306, provides less polar interactions between NRXβ1 and NL2 (Fig. 3F), thereby contributing to lower NL2 affinity. We note that the nonconserved residue NL2:Q475 (G500 in NL1) does not change conformation from apo to bound states (Fig. 3G). Therefore, the previously proposed mechanism of lower NL2 affinity due to Q475 steric hindrance is unlikely (*29*).

The above two differences at the NRXβ1 interaction site seem to underlie the differential affinities of NL1 to NL4 (Fig. 3H). NL1 (~10 nM) and NL4 (~100 nM) have higher affinities, and both have a Y at the NL2:H279 equivalent position. NL4 does not have the K at the NL1:K306 position, explaining its ~10-fold lower affinity than NL1. NL2 (~10 μM) and NL3 (~1 μM) both have lower affinities, and coincidentally, both have an H at the NL2:H279 equivalent position and obstruct the NRX pocket in the apo state (fig. S4C). This H explains the 10-fold lower affinity of NL3 than NL4. Further analysis of the available NL4 structure in the apo state (*28*) shows that NL2:H279 equivalent position (NL4:H236) adopts both conformations (fig. S4D). This suggests that the repulsion between neighboring HH (NL2/3) would consume energy to keep the NRX binding pocket open, lowering effective affinity (fig. S4E), while HY combination (NL1/4) does not have repulsion and thus shows higher affinities (fig. S4F). Consistent with this, substitution of NL2:H279Y and G282K increased NL2-NRXβ1 affinity by ~30-fold (Fig. 3I). Unfortunately, attempts to quantify contributions of H279Y and G282K individually were not successful because of lower yield and stability during purification. At the same time, since NL2 and NL3 are the same at both sites, this indicates that there is an unidentified mechanism for the further ~10× lower affinity of NL2. Structural information of NL3-NRXβ1, which is currently missing, and a detailed interrogation of each individual interaction, may provide a more systematic understanding.

Noncooperative binding may be an additional contributor. In the substoichiometric complex (Fig. 2, D and E, and fig. S2M, conf. 0), the bound and the unbound NL2 subunits exhibit different conformations, corresponding to the bound and apo states, respectively. This suggests a lack of cooperativity between the NRXβ1 binding sites of the two NL2 monomers, consistent with reported binding isotherms following single-site binding models with Hill coefficients of ~1 (*27*, *29*); here, we show the underlying structural reason. Being noncooperative prevents increased affinity through multiple binding events on the same NL2 dimer. These structural observations are consistent with reports that NL-NRXβ1 binding follows single-site interaction models (*27*, *44*).

### NRXβ1 and NL2 interaction is sufficient for tethering lipid membranes

**Fig. 4.**
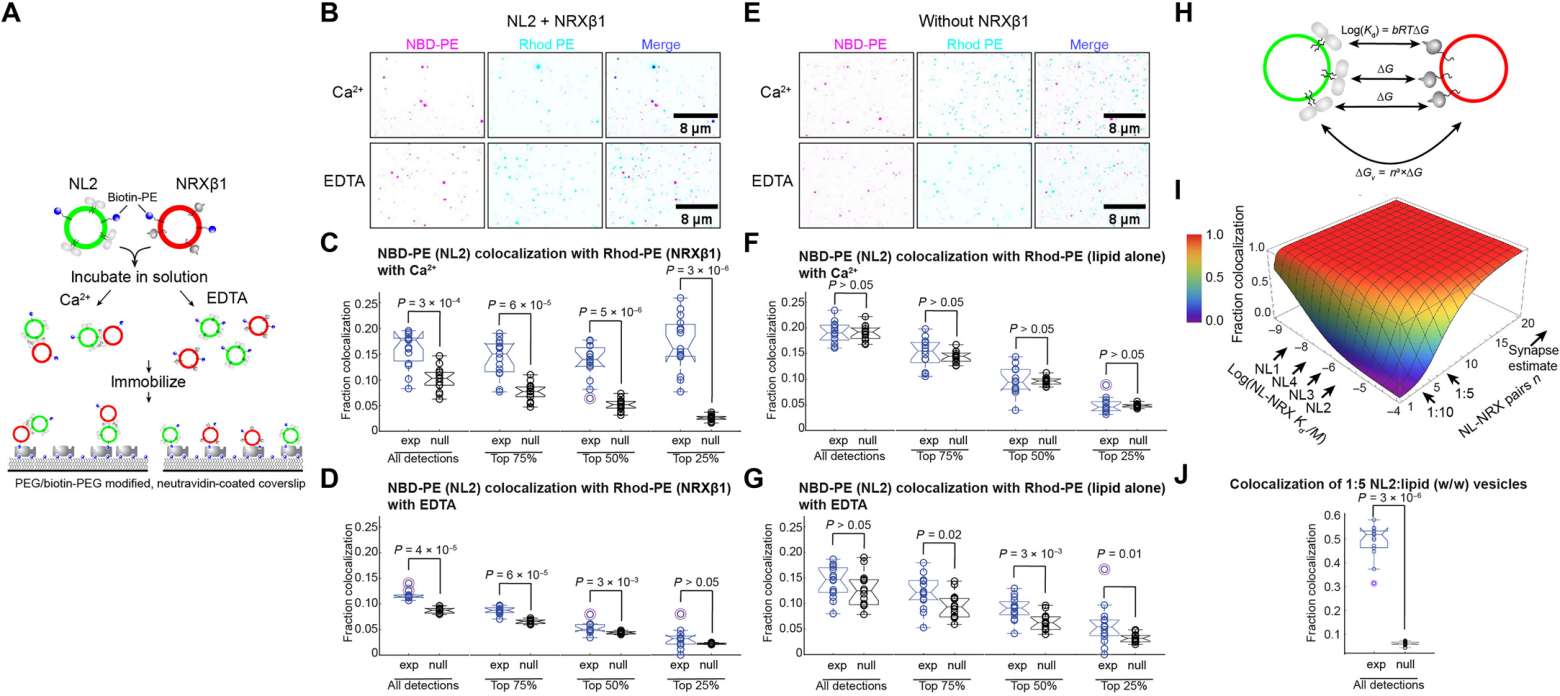
NRXβ1 and NL2 interaction tethers lipid membranes in reconstituted proteoliposomes. (**A**) Schematic of the vesicle colocalization assay. Nonlabeled wild-type NL2 and NRXβ1 were reconstituted in fluorescent vesicle containing NBD-PE (green) and Rhodamine-PE (red), respectively. (**B**) Representative micrographs with inverted contrast of NL2 (NBD-PE) and NRXβ1 (Rhod-PE) vesicles in the presence (top) and absence (bottom) of Ca^2+^. Object-based colocalization analysis for all vesicles and those with top 75, 50, and 25% fluorescence intensities in the presence of (**C**) Ca^2+^ or (**D**) EDTA. (**E**) Representative micrographs of NL2 (NBD-PE) and empty (Rhod-PE) vesicles in the presence (top) and absence (bottom) of Ca^2+^. Colocalization analysis in the presence of (**F**) Ca^2+^ or (**G**) EDTA. In (C), (D), (F), and (G), “exp” represents data, and “null” represents nullTR control (see Materials and Methods). Each circle represents one field of view, collectively summarized in a boxplot (the central mark, median; edges, 25th and 75th percentiles; dashed whiskers, most extreme inliers). Red circles indicate outliers (as deemed by the MATLAB “boxplot” function). Notch around median indicates the 95% confidence interval of the median. Number of fields of view: 15 (C), 12 (D), 13 (F), and 15 (G). (**H**) Illustration of vesicle tethering model. *n* pairs of NL-NRX interactions contribute to vesicle tethering with each binding free energy of Δ*G*, giving an effective total Δ*G_v_* = *n^a^* × Δ*G*, where constant *a* accounts for protein redistribution and other vesicle changes. (**I**) Plot of fraction colocalization (color coded as shown) as a function of individual NL-NRX *K*_d_ and the number of NL-NRX pairs *n*. NL1 to NL4 *K*_d_ values are indicated by arrows. Numbers of NL-NRX pairs corresponding to 1:10 and 1:5 NL2:lipid (w/w) reconstitution ratios and estimate in synapses are also indicated. (**J**) Colocalization analysis at 1:5 NL2:lipid (w/w) reconstitution ratio (2× protein concentration).

In each synapse, there are multiple adhesion proteins that contribute to the tethering of the two cellular membranes. Since the NL2-NRXβ1 interaction is the weakest among all NL-NRX pairs, it is unclear whether the NL2-NRXβ1 interaction alone is sufficient for tethering lipid membranes. To address this without complications from the undefined cellular environment, we used a total reconstitution system (Fig. 4).

Full-length wild-type (WT) NL2 and NRXβ1 were reconstituted separately into proteoliposomes supplemented with biotinylated lipids, as well as green (NBD-PE, NL2) and red (Rhod-PE, NRXβ1) fluorescent lipids (see Materials and Methods for details). Reconstituted proteoliposomes were incubated in solution to allow for unobstructed interaction and subsequently immobilization through biotin-neutravidin interaction on polyethylene glycol (PEG)–passivated glass coverslips (Fig. 4A). Total internal reflection microscopy (TIRF) was used to image immobilized vesicles, followed by distance-based colocalization analysis of detected vesicles (*45*, *46*).

Ca^2+^ promotes colocalization of NL2 with NRXβ1 proteoliposomes in a manner dependent on vesicles size (largely reflected as detected object intensity, given the diffraction-limited nature of most of the vesicles) (Fig. 4, B to D, and fig. S5, A to C). Visually, more colocalization is apparent in the presence of Ca^2+^, compared with no Ca^2+^ (EDTA as chelator) (Fig. 4B). When analyzing all detected vesicles, the difference between with and without Ca^2+^ is unclear (Fig. 4, C and D, “All detections”). However, when focusing on brighter detections (fig. S5A), Ca^2+^-dependent colocalization becomes increasingly notable while colocalization in its absence diminishes (Fig. 4, C and D). Vesicles with the top 25% intensities (i.e., intensities ≥75th percentile; fig. S5A) show clear, substantial colocalization in the presence of Ca^2+^ with a *P* value ~3 × 10^−6^ against the computational null control (see Materials and Methods for details) (*45*), compared with *P* value >0.05 without Ca^2+^ (Fig. 4, C and D, “Top 25%”). Analysis of the converse colocalization of NRXβ1 with NL2 vesicles yields similar outcomes (fig. S5, B and C). Since fluorescence is arising from fluorescent lipids at a given mole fraction, brighter detections represent larger vesicles within diffraction limit, which in turn contain more NL2 and NRXβ1 proteins on average for tethering membranes. Consistent with this, a higher ratio (1:5, w/w protein:lipid) reconstitution results in >0.6 colocalization (Fig. 4J, “All detections”). Since Ca^2+^ binds at the interaction interface between NL2 and NRXβ1 (Fig. 3A) and increases binding affinity (*22*, *27*), Ca^2+^ dependence further suggests a NL2-NRXβ1 interaction-dependent tethering.

Colocalization of vesicles requires the presence of both NL2 and NRXβ1 proteins. To evaluate protein-independent vesicle-vesicle interaction, we tested colocalization between NL2 proteoliposomes and lipid-alone vesicles that have the same lipid composition as NRXβ1 proteoliposomes, but without NRXβ1 (Fig. 4, E to G). No colocalization is obvious with or without Ca^2+^ (Fig. 4E). Quantitative analysis shows no significant colocalization (when tested against null controls), with *P* values mostly greater than 0.01, across all vesicle sizes (Fig. 4, F and G, and fig. S5, D and E). Clearly, a NL2-NRXβ1 interaction-dependent membrane tethering was observed in reconstituted lipid vesicles.

For a more comprehensive understanding of the avidity effect, we modeled vesicle tethering based on mass-action reactions (Fig. 4H). This model assumes *n* pairs of NL-NRX interactions, with individual *K*_d_ and binding free energy Δ*G*, resulting in a total tethering free energy Δ*G_v_ = n^a^ ×* Δ*G*, where *a* is a scaling factor to account for free energy costs in protein redistribution and lipid/vesicle conformation restrictions upon tethering. Equilibrium vesicle colocalization can be then evaluated on the basis of Δ*G_v_* (see Materials and Methods for more details).

Vesicle colocalization (tethering) is affected by individual NL-NRX *K*_d_ only when the number of NL-NRX pairs is low (Fig. 4I). At 1:10 NL2:lipid (w/w) reconstitution ratio (Fig. 4, C to G; see Materials and Methods), an average of *n* = 3.5 NL-NRX pairs contribute to the tethering between vesicles with a ~50-nm diameter. At this concentration, NL2 (6 μM) has ~0.1 colocalization fraction, and NL1 (10 nM) has over 0.9. By increasing the NL-NRX pairs twofold to *n* = 7, we observed over 0.5 colocalization for NL2 (Fig. 4J), as is predicted by the model (Fig. 4I). NLs and NRXs cluster at synapses with scaffolding proteins and receptors on the order of 100 to 1000 molecules per synapse (*47*, *48*). At *n* = 15 NL-NRX pairs, NL2 (6 μM) already results in over 0.97 colocalization, suggesting that NL2-NRX interaction likely plays an important role in tethering synaptic membranes through avidity effect.

### NRXβ1 and NL2 cluster at intercellular junctions

**Fig. 5.**
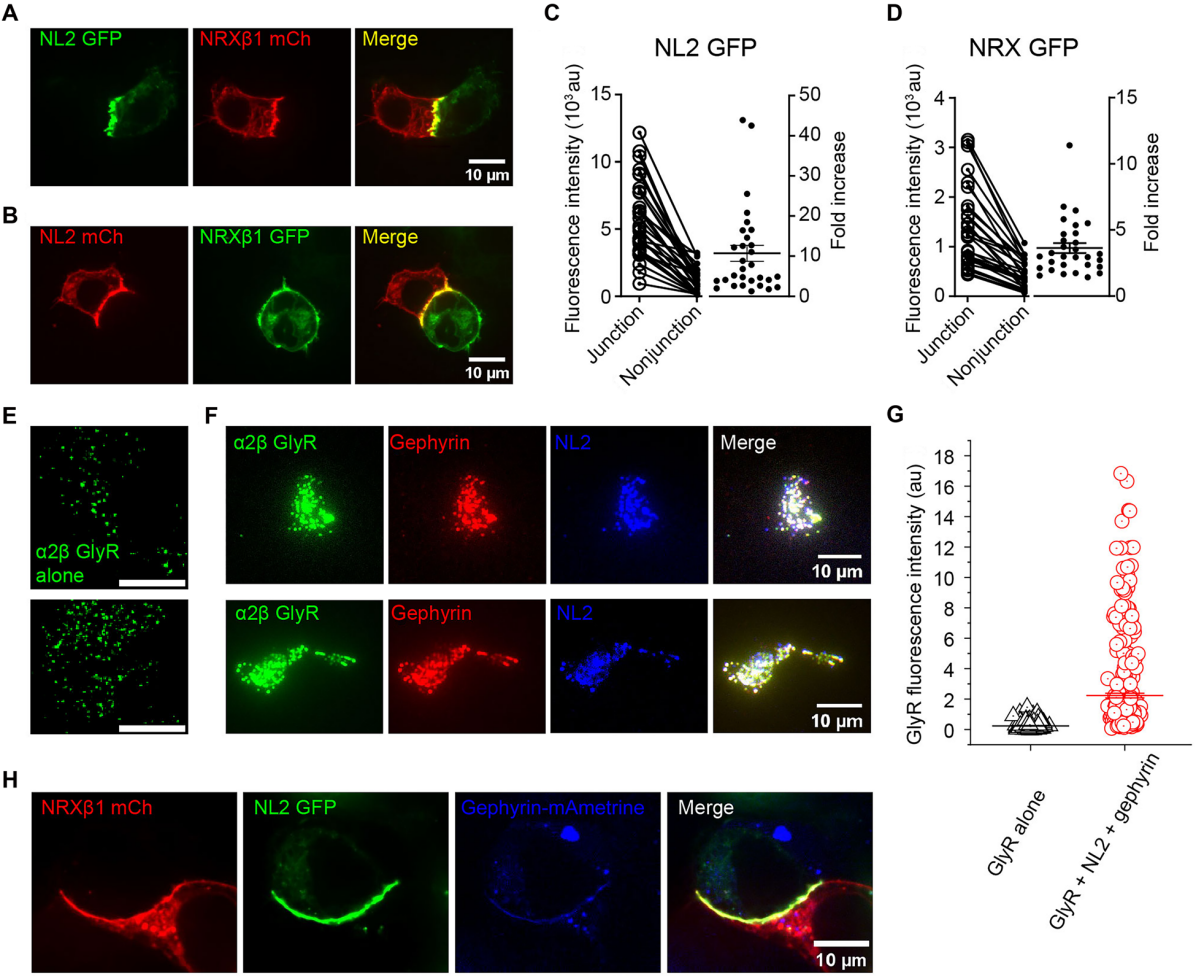
Quantification of NRXβ1 and NL2 at intercellular junctions. Representative confocal micrographs of (**A**) NL2-GFP and NRXβ1-mCherry and (**B**) NL2-mCherry and NRXβ1-GFP expressed in separated HEK293T cells and cultured together afterward. Quantification of (**C**) NL2-GFP and (**D**) NRXβ1-GFP fluorescence (*n* = 30 cells) at and outside of intercellular junctions. au, arbitrary units. Representative TIRF micrographs of unroofed cells expressing (**E**) α2β GlyR alone (green) or (**F**) together with gephyrin (red) and NL2 (blue), with merged image shown. (**G**) Fluorescence intensities of GlyR when expressed alone (black, *n* = 471 spots) or together with NL2 and gephyrin (red, *n* = 372 clusters). Bars represent means ± SEM. (**H**) Representative confocal micrographs of cocultured cells expressing NRXβ1-mCherry (red) or NL2-GFP (green) + gephyrin-mAmetrine (blue), respectively.

In neurons, NRXs and NLs are found clustered at opposing pre- and postsynaptic membranes (*8*, *11*). It is unclear whether locally concentrating NRXs and NLs requires other components in the synapse, including other synaptic adhesion molecules, intracellular scaffolding proteins, or receptors. To test this, we expressed enhanced green fluorescent protein (EGFP)– and mCherry-fused NL2 and NRXβ1 in separate human embryonic kidney (HEK) 293T cells and cultured them together. Apparently, NL2 and NRXβ1 are much more concentrated at intercellular contact sites (junctions) when compared to other regions of the plasma membrane, regardless of the fused fluorescent proteins (Fig. 5, A and B). Since HEK293T cells are incapable of forming synaptic specializations, concentration of NL2 and NRXβ1 at cellular interfaces does not require the formation of synapse.

We quantified both NL2 and NRXβ1 in their EGFP-fused form (Fig. 5, C and D) to avoid variables arising from differences in fluorescent protein fusions. Confocal slices were taken in the direction (largely) perpendicular to the cellular junctions. The fluorescence intensities increased an average of 10 ± 2–fold at junctions for NL2 (Fig. 5C) and 3.6 ± 0.4–fold for NRXβ1 (Fig. 5D). To obtain estimates of NL2 and NRXβ1 concentrations, we calibrated the fluorescence intensities against EGFP concentrations in solution (fig. S5H). The confocal volume was estimated on the basis of the numerical aperture and wavelength (see Materials and Methods for details), which provides sufficient accuracy at the higher concentrations (>100 nM) that we used (*49*). The number of NL2 dimers per square micrometer of junction membrane is largely in the range of 500 to 2000, while outside of junction, it is generally below 250. NRXβ1 is at lower concentrations, with ~400/μm^2^ at junction and ~100 outside the junction. As a comparison, we analyzed how NL1 concentrations change at the cellular junction (fig. S5, F and G). A 12 ± 2–fold increase of concentration was observed. This is very similar to that of NL2 (10 ± 2–fold), suggesting small contribution of difference in NRXβ1 affinity. This is likely a result of strong avidity effect arising from the large number of NL-NRX pairs at cellular junctions (Fig. 4I).

Clustering of NL2 and NRXβ1 at intercellular junctions suggests a mechanism for bridging two membranes despite low affinities; multiple NL2 and NRXβ1 interactions provide sufficient avidity. In addition, since the intracellular portion of NL2 and NRXβ1 is known to bind with synaptic scaffolding proteins and organizers in the post- and presynaptic specializations, respectively (*8*, *11*, *26*), clustering of NL2 and NRXβ1 likely serves as a local signal to coordinate synaptogenesis between two neurons through recruiting correspondent synaptic components.

### NL2 coclusters with gephyrin at the membrane and recruits α2β GlyR

NL2 is known to bind with the master scaffolding protein, gephyrin (*50*), which in turn binds with neurotransmitter receptors such as glycine (*51*, *52*) and γ-aminobutyric acid type A (GABA_A_) (*53*, *54*) receptors. Gephyrin has also been shown to bind to components of the cytoskeleton (*55**–**58*) and organize functional inhibitory synapses (*11*, *59**–**62*). To evaluate whether NL2 clustering signals the generation of functional synaptic specialization, we coexpressed heteromeric α2β GlyR, gephyrin, and NL2 in HEK293T cells. We performed unroofing to retain only the plasma membrane that was attached to the glass substrate and used TIRF microscopy to limit excitation depth (Fig. 5, E and F; see Materials and Methods for details). While α2β GlyR [green fluorescent protein (GFP) fusion at the β subunit; see Materials and Methods for details] alone forms diffraction-limited spots (Fig. 5E), coexpression with NL2 and gephyrin induced coclustering at the plasma membrane (Fig. 5F). Since these clusters contain functional α2β GlyR, they are poised to generate electrical activity upon binding with neurotransmitter glycine. Quantification shows a marked increase in the GlyR concentration within clusters, reaching more than 1000 receptors per square micrometer (based on ~0.3-μm point spread function diameter), and an average of more than 50 GlyRs/μm^2^. We further show that NL2 and gephyrin cocluster at the interface of cells expressing NRXβ1 (Fig. 5H), demonstrating that the clustering of NL2-NRX at the cellular junction recruits gephyrin to the membrane, likely serving as a signal for further synaptogenesis. It has also been reported that NL2 and gephyrin cocluster with another major inhibitory ionotropic receptor, the GABA_A_ receptor, indicating a universal role in inhibitory synapses (*50*). Local concentration of NRX upon binding with these clusters would signal the formation of cognate presynaptic specializations (*8*).

## DISCUSSION

Differential interaction between NL and NRX paralogs have been associated with the formation and specificity of synapses (*3*, *8*, *63*). NL1 and NL2 have 1000-fold difference in affinity to NRXβ1, with the mechanism for this being unclear. Through structural resolution using full-length proteins, we show that NL2-NRXβ1 form a stable complex despite low affinity (~10 μM). We identified two sites of NL that tune NRXβ1 affinity over a wide range. A flexible orientation between the TM and ECD, combined with structural flexibility that allows for variations in the orientation between the two monomers of the NL2 dimer, may facilitate adaptation to specific synapse geometry. We further found that the NL2-NRXβ1 low-affinity interaction is sufficient to tether lipid membranes through avidity effect. Clustering of NL2 and NRXβ1 at intercellular junctions help to both tether cellular membranes and recruit the scaffolding protein gephyrin, likely serving as signal to coordinate functional synapse generation.

**Fig. 6.**
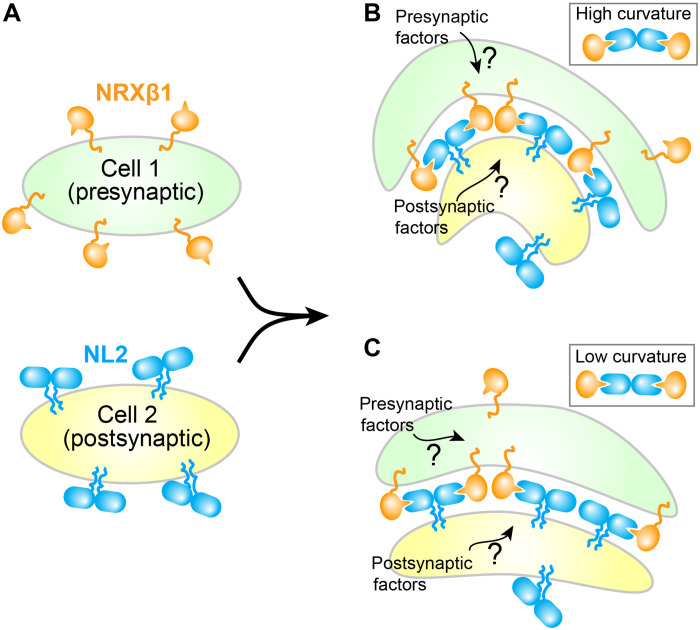
Illustration of NL2-NRXβ1 interaction at cellular junctions. (**A**) NL2 and NRXβ1 are randomly distributed on surface in the absence of intercellular contacts. ECD orientations with respect to membrane is flexible. Apo state NL2 adopts conformation that is incompatible with NRXβ1 binding. (**B** and **C**) NL2 and NRXβ1 cluster at cellular junctions, as full- or substoichiometric complexes. Despite weak interactions, multiple NL2-NRXβ1 binding provide sufficient avidity for tethering two membranes. Conformational variations between monomers of NL2 dimer may provide flexibility of large clusters at (B) high and (C) low membrane curvatures.

The above findings raise a tempting mechanism of how the NL2-NRXβ1 interaction contributes to spatial and chemical precision in the formation of functional synapse (Fig. 6). The plasma membranes of two cells come in proximity by chance and allow the formation of NL2-NRXβ1 adhesion complex (Fig. 6A). NL2 and NRXβ1 then cluster at the junction, providing more avidity. Flexible linkers between ECD and TM for both NL2 and NRXβ1, combined with variable orientation between NL2 monomers, make the adhesion complex amenable to membrane geometry (Fig. 6, B and C). Clustering of NL2 and NRXβ1 also results in locally concentrating their ICDs, which are known to interact with synaptic scaffolding proteins that in turn recruit factors necessary for synaptogenesis (*8*, *11*, *26*). Since NL and NRX only cluster at the interaction site, this mechanism allows spatial coordination between two cells. The specificity between NLs and NRXs, as well as between ICDs and respective scaffolding proteins, contributes to chemical specificity. NL2-NRXβ1 clustering likely initiates before synapse formation and serves as a synaptogenesis signal, consistent with its ability to induce synaptic formation in neurons (*64**–**66*)

The mechanism underlying NL2-NRXβ1 clustering is unclear. Since clusters are observed in HEK cells, they are unlikely to be dependent on any synapse-specific component. Clustering is less likely due to additional ECD interactions that were previously proposed on the basis of crystal contacts either (*31*), since such assembly implicates defined stoichiometry as well as observable interaction without crystallization, neither of which is supported by our observations. Instead, initial clustering/local concentrating may purely be a thermodynamic outcome—the NL2-NRXβ1 complex can only diffuse within the junction where membranes are sufficiently close, while NL2 or NRXβ1 alone does not have this limitation. This coincides with NRXβ1 being ~3.6-fold more concentrated at the junction and NL2 being ~10 ≈ 3.6^2^-fold more concentrated, as NL2 has two binding sites (thus double the energy) but NRXβ1 has only one. However, in cells, it might be much more complicated considering cross-interaction between other adhesion proteins and scaffolds, especially at cellular junctions where scaffolds in both cells may contribute to obstructed diffusion (*2*, *26*, *38*). In addition, the clustering of NL2 and gephyrin may also associate with specific lipids. In particular, gephyrin binds with collybistin (*58*), which in turn binds to phosphoinositides using the pleckstrin homology domain (*67*). NL2 clustering with gephyrin and collybistin has been shown to drive postsynaptic assembly (*50*). Clustering is thus likely regulated by a combination of the above mechanisms.

This work focused on homodimeric NLs; however, NL1/NL2 heterodimers have been clearly demonstrated (*18*, *68*, *69*). Because of the noncooperative nature of the NL-NRXβ1 interaction, apparent affinity should be a simple numeric average falling between that of NL1 and NL2. The composition of NLs may provide a fine-tuning mechanism for the average affinity. When the number of NL-NRX pairs exceeds 15, all NL combinations should provide sufficient avidity for membrane tethering. This is consistent with the observation that NL1 (fig. S5, F to H) concentrated similarly as NL2 at cellular junctions (Fig. 5, A to C). Although, the downstream signaling will likely be different depending on the NL type, which leads to different synaptic properties. When the NL-NRX pair numbers are small (conceivably at the initial phases of synaptogenesis), composition-dependent fine-tuning of affinities may play a crucial role in stabilizing synapse. The spatial organization of clusters containing different subtypes of NL and NRX may also differ and contribute to synaptic structure and function. Clearly, these aspects warrant future systematic investigation.

Tethering synaptic membranes through avidity effect from multiple weaker interactions in clusters offer advantages in dynamic regulation of synapses. A single interaction with nanomolar affinity correlates to ~1-min lifetime of the complex, assuming a 10^7^ s^−1^ M^−1^ diffusion-limited on-rate. Seven pairs of NL2-NRXβ1 interactions together also achieve an apparent nanomolar affinity, but with a complex lifetime in the 10-ms range. This allows NL2-NRXβ1–mediated tethers to be severed quickly by competing factors, such as MDGA1 (*32*, *33*) that binds NL2 with nanomolar affinities. This property also indicates that long-term stable tethering likely requires the involvement of other adhesion proteins. Clearly, how NL-NRX clusters contribute to different stages of synaptogenesis requires further investigation to understand the elegant construction and regulation of synapses.

## MATERIALS AND METHODS

### Cloning of NL2 and NRXβ1

The coding sequences of mouse NL2 and mouse NRXβ1 were amplified from pNICE-NL2(−) (Addgene item 15246) (*70*) and pNICE-LAP-NRX-β1 (Addgene item 42575) (*71*), respectively. The NL2 sequence does not contain the splice insert at site A (missing G153-T169 in Uniprot no. Q69ZK9) (*35*), while the NRXβ1 does not contain the insert at splice site 4 (missing N202-R232 of Uniprot P0DI97-1). They were subsequently cloned into BacMam vectors for expression in HEK293T cells (*72*, *73*). The signal peptide of NL2 was replaced with that of NL1 followed by a hemagglutinin (HA) tag (see fig. S1). Both proteins were constructed as an EGFP fusion at the C terminus for affinity purification using anti-GFP antibodies (*74*). A PreScission protease (PPX) site was included before the EGFP (see fig. S1A).

### Protein expression and purification for NL2 apo structure

A vector containing NL2 was transformed into DH10BacY competent cells (Geneva Biotech) to produce bacmids. Sf9 cells were transfected (Invitrogen) with the bacmids and then amplified in Insect-XPRESS medium (BioWhitaker). Protein expression was done on a large scale using 2 liters of HEK293T cells grown in Freestyle 293 medium (Gibco) containing 1% fetal bovine serum and 1% penicillin-streptomycin. Cells were infected with virus at a density of 2.0 × 10^6^ cells/ml, induced using 10 mM sodium butyrate 20 hours after infection, and grown for a total of 72 hours. After harvesting, cells were lysed using 10 mM Hepes-Na (pH 7.5), 50 mM NaCl, 1 mM MgCl_2_, and 0.5 mM CaCl_2_. The protein was extracted using 0.5% lauryl maltose neopentyl glycol (LMNG) in a 20 mM Hepes-Na (pH 7.5), 200 mM NaCl, 1 mM MgCl_2_, and 0.5 mM CaCl_2_ buffer for 1 hour at 4°C. Protein was purified using GFP-nanobody resin and eluted by PPX digestion and then further purified using size exclusion chromatography using a Superose 6 increase 10/300 GL (GE) column in a buffer containing 20 mM Hepes-Na (pH 7.5), 150 mM NaCl, 1 mM MgCl_2_, 0.5 mM CaCl_2_, 0.06% digitonin, and brain polar lipid extract (0.05 mg/ml). Peak fractions were collected and concentrated.

### Expression and purification of full-length NL2 and NRXβ1

Virus containing NRXβ1 was produced in like fashion to NL2. Protein expression for each protein was done separately using the same method. HEK293T cells were infected with virus at a density of 2.0 × 10^6^ cells/ml, induced using 10 mM sodium butyrate 20 hours after infection, and grown for a total of 72 hours. After harvesting, cells were lysed using 10 mM Hepes-Na (pH 7.5), 50 mM NaCl, 1 mM MgCl_2_, and 0.5 mM CaCl_2_. The protein was extracted using 0.5% LMNG in a 20 mM Hepes-Na (pH 7.5), 200 mM NaCl, 1 mM MgCl_2_, and 0.5 mM CaCl_2_ buffer for 1 hour at 4°C. Protein was purified using GFP-nanobody resin and eluted by PPX digestion and then further purified using size exclusion chromatography using a Superose 6 increase 10/300 GL (GE) column in a 20 mM Hepes-Na (pH 7.5), 150 mM NaCl, 1 mM MgCl_2_, 0.5 mM CaCl_2_, and 0.05% LMNG buffer. Peak fractions were pooled together and concentrated using an Amicon Ultra-4 100-kDa molecular weight–cutoff centrifugal filter (Millipore).

### Cryo-EM sample preparation

NL2 and NRXβ1 were purified and concentrated separately. They were then mixed at a one-to-one molar ratio for a final concentration of 1.8 mg/ml of NL2 and 1.0 mg/ml of NRXβ1 and incubated on ice for 10 min. This corresponded to ~20 μM (monomer) protein each, roughly 3× *K*_d_ (apparent *K*_d_ ~ 6 μM; see Fig. 3H). This concentration was chosen, on the basis of trial and error, to ensure that it does not induce aggregation and at the same time is high enough to drive complex formation. Samples (3.5 μl) were applied to each glow-discharged Quantifoil R1.2/1.3 400-mesh gold holey carbon grid (Quantifoil, Micro Tools GmbH, Germany), blotted under 100% humidity at 4°C and vitrified in liquid ethane using a Mark IV Vitrobot (FEI).

### Cryo-EM data acquisition and processing

Micrographs were collected using a Titan Krios microscope (Thermo Fisher Scientific) with a K3 Summit direct electron detector (Gatan) operating at 300 kV. SerialEM was used for automated data acquisition with a GIF-Quantum energy filter set to a slit width of 20 eV. Images were recorded in the super-resolution counting mode with the pixel size of 0.415 Å. Micrographs were dose fractioned into 50 frames with a dose rate of 1.8 e^−^/Å per frame for NL2 apo and 1.4 e^−^/Å per frame for the NL2-NRXβ1 complex. Four thousand nine hundred eighty-eight and 6615 movies were collected for NL2 apo and the NL2-NRXβ1 complex, respectively.

Motioncorr2 (*75*) was used for Fourier truncation (twofold binning, 0.83-Å pixel size afterward), motion correction, and dose weighting of the movie frames, with subsequent Contrast Transfer Function (CTF) correction performed using CTFFIND 4 program (*76*). Image processing steps were carried out in RELION 3 (*77*) and cryoSparc (*78*, *79*), as illustrated in fig. S2. Initially particle picking using the Laplacian-of-Gaussian blobs allowed two-dimensional (2D) classification to obtain good class averages, which were used for templates for reference-based autopicking. The resulting particles were cleaned up with further rounds of 2D classifications. Good 2D classes were used to generate initial 3D models and subsequent 3D classification and refinement as detailed below.

For NL2 apo, 3D heterogeneous refinement with three classes in cryoSparc (*78*) resulted in one good class, containing 77,577 particles. This class was further subjected to Non-Uniform refinement (*79*) and reached an overall resolution of ~3.3 Å.

For the NL2-NRXβ1 complex, 3D classification into eight classes resulted in two good classes, class 2 and class 5, with class 2 having inverted projection. After flipping Z for class 2 using Chimera software (*80*) and further 3D classification for both class 2 and class 5 (fig. S2I), one substoichiometric (one NRXβ1: one NL2 dimer, conf. 0), and three full-stoichiometric (two NRXβ1: one NL2 dimer, conf. 1 to 3) complex structures were identified. Particles of each structure were CTF refined and Bayesian polished in RELION after Non-Uniform refinement in cryoSparc. A final round of Non-Uniform refined with polished particles resulted in the final density maps with overall resolutions of 3.9 Å for conf. 0 and 3.2 Å for conf. 1 to 3 (fig. S2, J to L). Good local resolutions extending beyond 2.5 Å were observed in NL2, while those of NRXβ1 are more moderate, especially in parts further away from NL2 (fig. S2M). Resolutions were estimated by applying a soft mask around the protein densities with the Fourier shell correlation 0.143 criterion. Local resolutions were calculated using Resmap (*81*).

### Model building and refinement

The model of apo NL2 was made by aligning the NL2 structure [Protein Data Bank (PDB) ID: 3BL8] to the density map using Chimera (*80*). The model was then adjusted, and the amino acid residues were substituted manually using Coot (*82*). The model was refined using the real-space refinement program in PHENIX (*83*). The final NL2 model begins at R40 and shows all amino acid residues except the following due to low map density: chain A: E151-S174, D553-N563, and ends at L608; chain B: E151-S174, I557-P562, and ends at N610. For the NL2-NRXβ1 complexes, each monomer (PDB ID: 3BIW) was fitted into experimental maps using Chimera, manually adjusted using Coot, and refined in PHENIX (see table S1 for statistics) similarly to the NL2 Apo model. Statistics of cryo-EM data processing can be found in table S1. For conf. 0, the NL2 subunit begins at E39 for chain A and R40 for chain B and shows all residues except for as follows due to low map density: chain A: D152-G175, F557-K561, and ends at N610; chain B: D152-G175, F557-K561, and ends at H609. The NRXβ1 subunit begins at A83 and shows all residues except for as follows due to low map density: E162-I168, R202-L231, and ends at G289. For conf. 1, both NL2 chains start at R40 and end at L608, with no modeling for E151-D173, T555-N563 for chain A, and E151-D173 and D554-N563 for chain B. For conf. 2, both NL2 chains start at R40 and end at L608, with no building for E151-D173 and P552-P562 for chain A and E151-D173 and K556-P562 for chain B. For conf. 3, the NL2 subunit begins at R40 for both chains A and B. Omissions due to low density for the NL2 subunit are as follows: T150-S174 for chain A, T150-D173 for chain B, D554-N563 for both chains, and both chains end at L608. The NRXβ1 subunit chains for conf. 1, 2, and 3 all have the same missing amino acid residues, with the chains beginning at A83 and missing R202-L231, and ending at L289.

### mPEG glass preparation

Glass coverslips were cleaned by soaking in 100 ml of Piranha solution for 90 min, sonicating for 10 s at the start and every 45 min after. The slides were rinsed with Mili-Q H_2_O five times. The clean slides were etched using 1 M KOH and soaked for 30 min, sonicating for 1 min at the start and every 15 min after. The coverslips were then rinsed with Mili-Q H_2_O and dried using filtered air. The coverslips were then preheated to 90°C, and 10 μl of 25% mPEG-sliane 5k with 1% biotin mPEG-silane 5k in dimethyl sulfoxide was added to the coverslip. A second coverslip was added to coat the whole area. The coverslips were then incubated at 90°C for 30 min, rinsed five times with Mili-Q H_2_O, and dried using filtered air. The slides were stored at −20°C for later use.

### Proteoliposome reconstitution and TIRF imaging

A 90:10:0.1:0.1 1-palmitoyl-2-oleoyl-glycero-3-phosphocholine (POPC): 1-palmitoyl-2-oleoyl-*sn*-glycero-3-phospho-l-serine (POPS): 1,2-dioleoyl-*sn*-glycero-3-phosphoethanolamine-*N*-(cap biotinyl) (biotin-PE):1,2-dioleoyl-*sn*-glycero-3-phosphoethanolamine-*N*-(7-nitro-2-1,3-benzoxadiazol-4-yl) (NBD-PE) (Avanti) lipid mixture was dried using argon gas and placed in a vacuum desiccator overnight. After drying, lipids were resuspended in Mili-Q H_2_O at 20 mg/ml and sonicated until clear and then solubilized with 0.5% *n*-dodecyl-β-ᴅ-maltoside (DDM). The lipids were added to purified NL2 protein at a ratio of 1:10 (1:5 for 2× concentration in Fig. 4J) protein to lipid. This mixture was then diluted down with a 40 mM Hepes-Na (pH 7.5), 300 mM NaCl buffer to a final concentration of 10 mg/ml. Bio-Beads SM-2 (Bio-Rad) were added to the mixture to remove detergent, and the mixture was rotated at 4°C. The Bio-Beads were changed 2× every 3 hours and changed 1× overnight. For NRXβ1, a second lipid mixture was made in a similar manner with the composition being 90:10:0.1:0.1 POPC:POPS:biotin-PE: 1,2-41dioleoyl-*sn*-glycero-3-phosphoethanolamine-*N*-(lissamine rhodamine B sulfonyl) (Rhod-PE), and the protein was reconstituted for a ratio of 1:20 (1:10 for 2× concentration in Fig. 4J) protein to lipid. The reconstituted proteins were mixed together at a concentration of 0.1 mg/ml and incubated for 30 min and then further diluted for a final concentration of 0.33 μg/ml using bovine serum albumin (BSA) (2 mg/ml), 20 mM Hepes-Na (pH 7.5), and 150 mM NaCl (dilution buffer). To each clean coverslip, 15 μl of 280 nM NeutrAvidin in BSA (5 mg/ml), 20 mM Hepes-Na (pH 7.5), and 150 mM NaCl was added and incubated at room temperature for 15 min. The coverslips were then washed five times with 1 ml of 20 mM Hepes-Na (pH 7.5), 150 mM NaCl. Then, 15 μl of the reconstituted vesicles was added to the coverslip and incubated at room temperature for 15 min and then rinsed with the dilution buffer nine times. The slides were then imaged using TIRF. This imaging was achieved using an in-house built prism-type system using the Gem 488-nm laser (Laser Quantum) and the Gem 560-nm laser (Laser Quantum) and components from Thorlabs at 10% power with an exposure time of 200 ms. A Leica DM6 FS microscope equipped with a 60× 1.2–numerical aperture (NA) water immersion objective and a Hamamatsu flash 4.0 V3 camera was used for imaging. Images were collected using a PC running Metamorph (Molecular Devices).

### Vesicle detection and colocalization analysis

Imaged vesicles were detected and localized using the “gaussian mixture-model fitting” algorithm in u-track (*84*) (version 2.2.1; https://github.com/DanuserLab/u-track), using the iterative mixture-model fitting option. For both channels, the Gaussian SD was taken as 1.1 pixel (113 nm), and the α-values for the residuals test, amplitude, test and distance test were respectively set to 0.05, 0.1, and 0.05. The α-value for local maxima detection was set to 0.05 for the 560-nm channel (NRXβ1) and 0.1 for the 488-nm channel (NL2). Visual inspection confirmed that these detection parameters detected all objects, both dim and bright, with minimal false positives or false negatives.

Object-based colocalization analysis was then performed on the detected vesicles, on the basis of the nearest-neighbor distances between detected objects in the two channels, as described (*45*), using a colocalization radius of 3 pixels (code available at https://github.com/kjaqaman/conditionalColoc). Colocalization analysis was performed on either all vesicles (all circles in fig. S4A) or on those with intensities in the top 75% (green, cyan, and magenta circles in fig. S4A), in the top 50% (green and cyan circles in fig. S4A), or in the top 25% (green circles in fig. S4A). To assess the significance of any measured colocalization, i.e., the likelihood of it happening by chance, a “nullTR” colocalization measure (computational null control) was calculated for each sample, where the objects in one of the two channels were replaced by points on a grid. The real data colocalization measure was then compared to the nullTR measure using a Wilcoxon rank sum test to compare medians, yielding the reported *P* values.

### Modeling of vesicle tethering through avidity effect

The extent at which colocalization of vesicles is determined by the *K*_d_ of individual NL-NRX interactions and the number of NL-NRX pairs can be modeled on the basis of mass-action binding (Fig. 4H). Let the free energy change of single NRX-NL interaction be Δ*G*. Assuming that *n* pairs of NL-NRX interactions between two vesicles contribute to tethering, with a scaling factor *a* to account for free energy cost in protein redistribution and lipid/vesicle conformation restrictions upon tethering, the effective free energy of vesicle tethering Δ*G*_v_ can be expressed as

$$∆G_{v}=n^{a}\times ∆G$$ (1)

The equilibrium *K*_d_ of a single binding reaction is related to the free energy change Δ*G* upon binding as in

$$\text{bRT}\text{Log}\left(K_{d}\right)=∆G$$ (2)

where *b* is a constant to account for the base of logarithm, *R* is the gas constant, and *T* is the absolute temperature. Equation 2 can be rearranged as

$$K_{d}=e^{\left(∆G/\text{RTb}\right)}$$ (3)

Assuming that vesicle tethering is governed by mass action, equilibrium dissociation constant of vesicles *K*_dv_ can be expressed as

$$\begin{array}{cc} K_{\text{dv}} & =(V_{\text{NL}}-V_{\text{coloc}})\times \left(V_{\text{NRX}}-V_{\text{coloc}}\right)/V_{\text{coloc}}\\ & =e^{∆G_{v}/\text{RTb}}=e^{\left(n^{a}\times ∆G/\text{RTb}\right)}=e^{\left[n^{a}\times \text{Log}\left(K_{d}\right)\right]} \end{array}$$ (4)

where *V*_NL_, *V*_NRX_, and *V*_coloc_ are vesicle concentrations of total NL, total NRX, and colocalized (tethered) NL and NRX. Colocalization fraction *f* = *V*_coloc_*/V*_NL_ was resolved and plotted using Mathematica (Wolfram).

$$f=\frac{{K_{d}}^{n^{a}}+V_{\text{NRX}}+V_{\text{NL}}-\sqrt{{\left(-{K_{d}}^{n^{a}}-V_{\text{NRX}}-V_{\text{NL}}\right)}^{2}-4V_{\text{NRX}}V_{\text{NL}}}}{2V_{\text{NL}}}$$ (5)

The model recapitulated both 1:10 and 1:5 NL2:lipid reconstitution colocalizations using an *a* value of 0.48.

### Cell imaging using confocal microscopy

Two plates of HEK293T cells were transfected separately with NL2-EGFP and NRXβ1-mCherry or NL2-mCherry and NRXβ1-EGFP for the NL2 and NRXβ1-only assay using a Lipofectamine 3000 Transfection Kit (Invitrogen) and grown overnight. For the NL2, NRXβ, and gephyrin assay, one plate was transfected with both NL2-EGFP and gephyrin-mAmetrine, and a second plate was transfected with NRXβ1-EGFP. For the NL1 assay, one plate was transfected with NL1-EGFP, and the second plate was transfected with NRXβ1-mCherry. The cells were then mixed on a collagen-treated glass-bottom dish (Mattek) and allowed to grow for 3 hours at 37°C. The cells were then fixed using a 4% paraformaldehyde (PFA) (Electron Microscopy Sciences) in Dulbecco’s Phosphate Buffered Saline (DPBS; Gibco) for 15 min at room temperature. After fixing, the cells were imaged using a Nikon Ti2E microscope equipped with a Yokogawa CSU-X1 spinning disk using a 60× oil immersion objective with the appropriate filter set, and images were collected with 488- and 561-nm laser at 20% power. NL1 confocal images were collected using a Leica DMI6000 microscope, with a Yokogawa CSU-X1 spinning disk confocal scanner, a Hamamatsu ImagEMX2 electron-multiplying charge-coupled device camera, a Leica 63× 1.4-NA oil immersion objective, and a 405/488/561/647-nm Laser Quad Band Set filter cube (Chroma). Images were processed using ImageJ (see below).

### Quantification of fluorescence intensity

Intensity analysis was performed using ImageJ. For the NL2 images, the background of the image was determined at areas with no cell and subtracted from measurements. For quantification of the NL1 junction images, the background was subtracted using the rolling ball background subtract function on ImageJ with a radius of 50 pixels (0.206-μm pixel size). Using the Plot Profile function on ImageJ, we plotted the junction fluorescence intensity of the two cells in the EGFP channel and used one-third of the measured peak value as the threshold for region of interest (ROI), within which mean fluorescence intensities were calculated. For the same cell, we used a similar method to quantify fluorescence intensity of the nonjunction membrane. The junction intensity was divided by the nonjunction intensity to give us the fold difference. To estimate protein concentration, the fluorescence intensities were measured at different concentrations (0, 0.1, 1, 3, and 9 μM) of soluble EGFP proteins. Number of molecules contributing to fluorescence was calculated using $N=\left[\text{GFP}\right]\times V_{c}\times N_{A}$, where *V*_c_ is the effective confocal volume and *N*_A_ is Avogadro’s number (6.02 × 10^23^). *V*_c_ is estimated using $V_{c}=\pi^{\frac{3}{2}}r_{\mathrm{xy}}^{2}r_{z}$, where $r_{\mathrm{xy}}=\frac{0.61\;\lambda }{\text{NA}}$ and $r_{z}=\frac{2\;n\;\lambda }{{\text{NA}}^{2}}$ are the diffraction-limited radii in *x*-*y* and *z* directions, respectively. λ (excitation wavelength) is 488 nm, *n* (sample refractive index) is 1.33, and NA (objective numerical aperture) is 1.4.

### Purification of NRX ECD

NRXβ1 ECD His-tag purification was performed by transforming the NRXβ1 ECD (amino acids 81 to 311) DNA into BL21 competent *E. coli* cells and grown into a large-scale 1-liter prep. The cells were grown to a density of 0.8 to 1.0 optical density and induced with 0.2 mM isopropyl-β-d-thiogalactopyranoside and then grown at 16°C for 24 hours. Once the cells were harvested, they were resuspended into binding buffer [20 mM tris, (pH 8.0) and 500 mM NaCl] and then sonicated for 1 min three times with stirring at 4°C for 5 min in between each sonication. The cells were then centrifuged, and the supernatant was loaded on to Ni–nitrilotriacetic acid resin (Biosciences). The protein-bound resin was washed with 50 ml of binding buffer and then washed with 50 ml of 20 mM tris, (pH 8.0), 500 mM NaCl, and 40 mM imidazole. The protein was then eluted off the resin using 50 ml of 20 mM tris (pH 8.0), 500 mM NaCl, and 500 mM imidazole. The protein was concentrated and further purified using size exclusion chromatography using an ENrich Sec 70 10X300 Column (Bio-Rad).

### Fluorescent labeling of NRX ECD

NRXβ1 ECD (0.5 mg) was diluted in labeling buffer (20 mM Hepes, 150 mM NaCl, and 0.05% LMNG) and fluorescently labeled with 200 mM Alexa Fluorophore 488-NHS by overnight incubation at 4°C. The protein dye mixture was then desalted in a prepacked Sephadex G-25 M column (PD-10, GE Healthcare) using labeling buffer. Labeled protein was concentrated and further purified using size exclusion chromatography using the SEC 70 column in the labeling buffer.

### Fluorescent polarization

Fluorescent polarization was measured for NRXβ1 ECD labeled with Alexa Fluorophore 488, during titration of either NL2 WT or NL2mut (H279Y G282K). Using a 96-well Optical Bottom PolymerBase plate (Thermo Fisher Scientific) a triplicate was performed for each NL2 WT and NL2mut. The titration was performed in 20 mM Hepes (pH 7.5), 150 mM NaCl, 1 mM MgCl_2_, and 0.5 mM CaCl_2_ with 8 nM NRXβ1 ECD–AF488. The NL2/NL2mut concentrations were 15 nM, 30 nM, 62.5 nM, 125 nM, 250 nM, 500 nM, 1 μM, 2 μM, 4 μM, and 8 μM. The plate was imaged using Spark Multimode Microplate Reader (TECAN) Fluorescent polarization methods with an excitation wavelength of 490 and an emission wavelength of 540.

### Cotransfection and TIRF imaging of clusters

Slip-rite cover glass (no. 1, 22 mm by 22 mm) (Thermo Fisher Scientific) was cleaned using sulfuric acid for 30 min, then rinsed with Mili-Q water three times, and dried in a sterile hood for 1 hour. The coverslips were then placed into a 35-mm dish, coated with collagen (100 μg/ml), and incubated for 1 hour at 37°C. They were then rinsed with phosphate-buffered saline (PBS) three times and dried in sterile hood for 1 hour. The plates containing the coverslips were then seeded with 0.4 million of HEK293T cells and transfected with NL2 containing a Halo tag on the C terminus, GPHN (residues 2 to 736 with N-terminal mCherry tag), GlyRα2em, and GlyRβem at a ratio of 2:2:1:1 using a Lipofectamine 3000 Transfection Kit (Invitrogen) and grown for 20 hours (*2*). GlyRα2em and GlyRβem have been described before (*73*). Briefly, the GlyRα2em subunit has an oxidized glutathione substitution at residue Q317-K381; the GlyRβem has two modifications; residue N334-N377 was replaced by GGSSAAA-monomeric EGFP (mEGFP)-SGSGSG, and at the N terminus, a PA-tag (GVAMPGAEDDVV) (*85*) and a PPX site (LEVLFQ/GP) (*86*) were inserted. The control plates containing GlyR alone were transfected with GlyRα2em and GlyRβem at a ratio of 1:3. The cells were then washed at room temperature three times with PBS and then sonicated at 20% power for 1 s using PBS supplemented with 2% PFA. After sonication, the cells were washed with PBS and incubated at room temperature for 15 min in PBS containing 4% PFA. The cells were then washed with PBS three times for 5 min each. To label the NL2, the cells were blocked for 15 min using PBS with 1% BSA, then the primary antibody, HA-tag C29F3 rabbit mAb (Cell Signaling Technology), was added at a ratio of 1:2000, and the cells were incubated for 1 hour at room temperature. The cells were then rinsed with PBS, and the secondary antibody, Goat Anti-Rabbit IgG, Highly Cross-Adsorbed, Labeled with CF660C dye (BIOTIUM), was added to the cells at a concentration of 1 μg/ml and incubated at room temperature for 1 hour. The cells were then rinsed once more with PBS, and the coverslip was then imaged using the same TIRF imaging methods as described above.

### Quantification of TIRF micrographs

ImageJ was used for quantification. To quantify the florescent intensity of the GlyR-only images, the ImageJ plugin track mate was used to detect spots as ROI points. The fluorescence intensity inside each ROI were quantified. The background was estimated at areas with no detections and then subtracted from those measurements. To measure the fluorescence intensity of the GlyR clusters, the background was subtracted from the images using the rolling ball background subtract function on ImageJ with a radius of 50 pixels (0.206-μm pixel size). Clusters were then detected by using the find maxima function (ImageJ), using strict, exclude edge maxiam, and a prominence of 200, with an output type of maxima within tolerance. ROIs were created around these maxima, and fluorescence intensity was quantified. Histograms were generated using the OriginPro program.
