# Supplementary material for: Weaker neuroligin 2–neurexin β1 interaction tethers membranes and recruits gephyrin at membrane junctions through clustering
Source: Sci Adv. 2026 Mar 13;12(11):eads9732. doi: 10.1126/sciadv.ads9732 (PMC12985673; doi:10.1126/sciadv.ads9732)
Supplement: Supplementary file 1 — Figs. S1 to S5 Table S1 [file sciadv.ads9732_sm.pdf]

Science Advances
AAAS

Supplementary Materials for

Weaker neuroligin 2–neurexin β1 interaction tethers membranes and recruits gephyrin at membrane junctions through clustering

Robbie Boyd *et al.*

Corresponding author: Weiwei Wang, weiwei.wang@utsouthwestern.edu

Sci. Adv. **12**, eads9732 (2026)
DOI: 10.1126/sciadv.ads9732

This PDF file includes:

Figs. S1 to S5
Table S1

Supplementary Materials

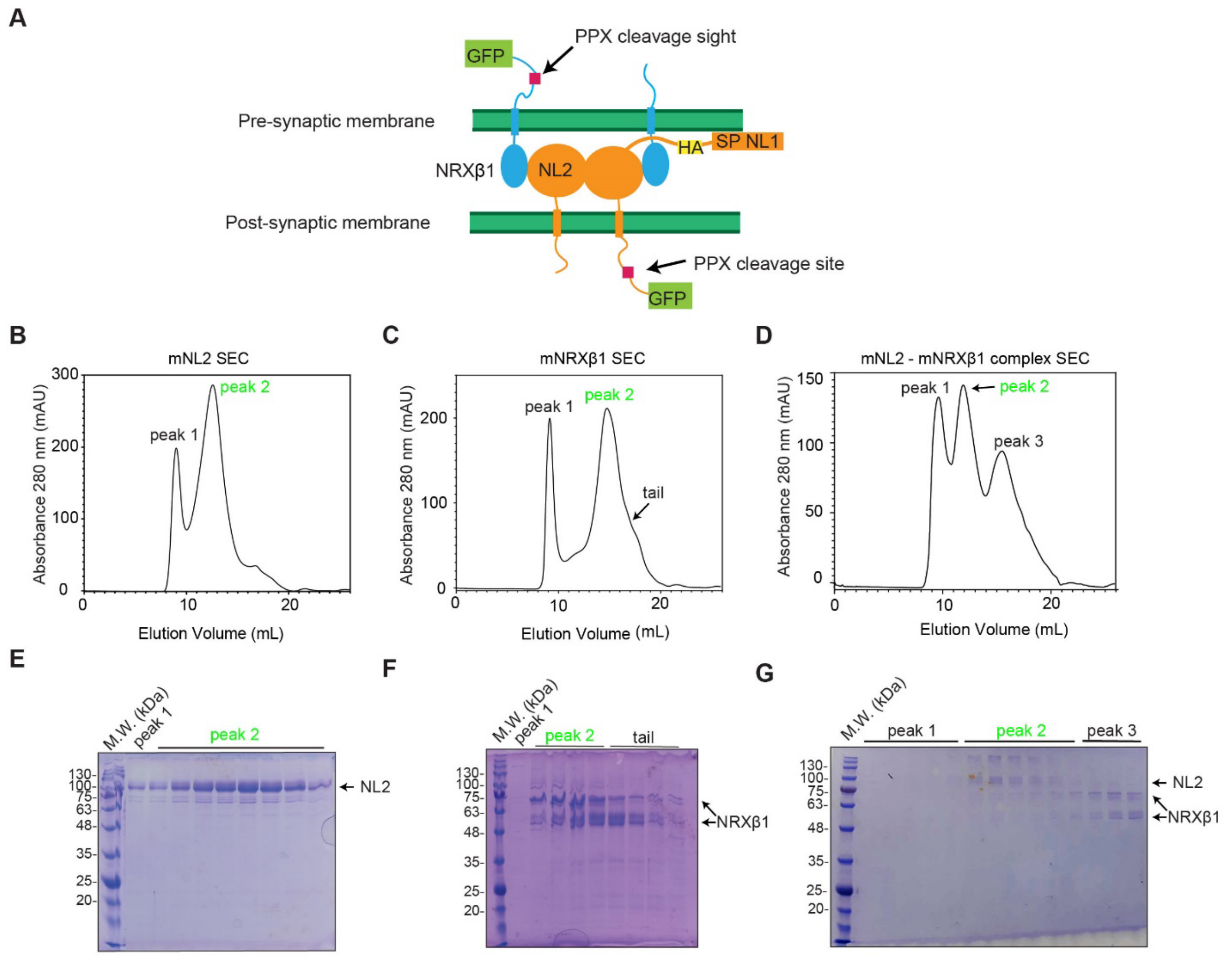

Figure S1. Expression and purification of NL2 and NRXβ1. (A) Illustration of full-length NL2 and NRXβ1, with signal sequence, tags, and PPX cleavage sites shown. GFP tag allowed purification using GFP-nanobody resin. (B-D) SEC elution profiles of respective proteins, with peak 1 representing the void volume, and peak 2 being the desired protein/complex. (E-G) SDS PAGE of respective SEC fractions.

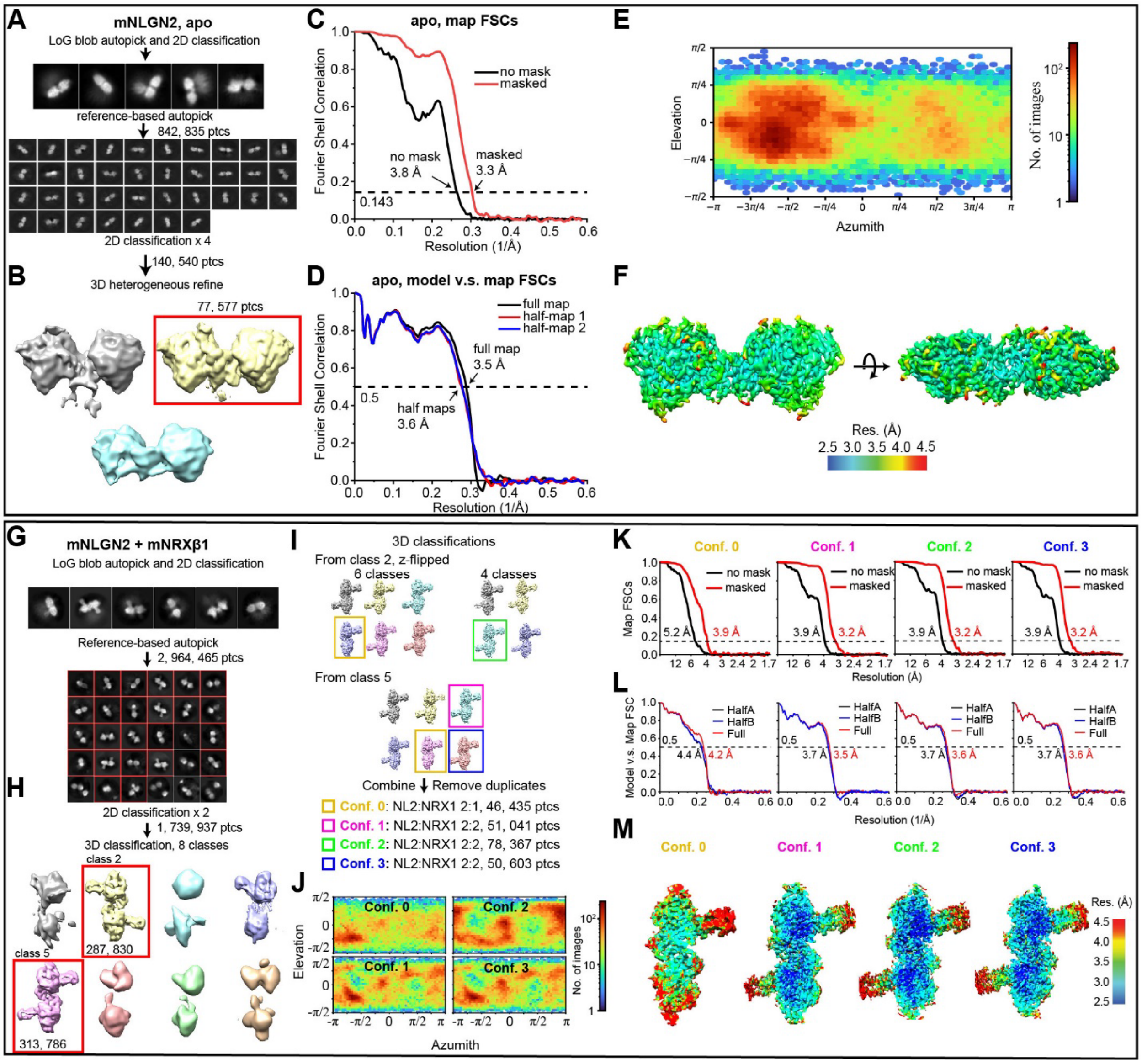

Figure S2. **Overview of cryo-EM data processing. (A-F**) Processing of NL2 apo dataset. (**A**) Particle picking and 2D classification. (**B**) Classification into three 3D classes resulted in one good class containing 77, 577 particles. Fourier-Shell Correlations (FSC) between (**C**) two half maps and (**D**) atomic model and maps, with resolution at FSC of 0.143 (between maps) and 0.5 (model v.s. maps) indicated. (**E**) Angular distribution of particles in final refinement. (**F**) Density map colored according to local resolutions view from side and top. **(G-M**) Processing of mNL2-mNRXβ1 dataset. (**G**) Particle picking and 2D classification. Red rectangles indicate selected classes. (**H**) Classification into eight 3D classes resulted in two good class (red rectangles) with

the numbers of particles in each class indicated. (**I**) Further 3D classification yielded good resolution maps with distinct conformations (yellow, purple, green and blue respectively corresponds to Conf. 0, 1, 2, 3). (**J**) Angular distributions of particles in final refinement for each conformation. FSCs between (**K**) two half maps and (**L**) atomic model and maps, with resolutions at 0.143 FSC (between maps) and 0.5 FSC (model v.s. maps) indicated. (**M**) Density maps sliced through center, viewed from top and colored according to local resolutions. Local resolutions were estimated using ResMap 1.1.4.

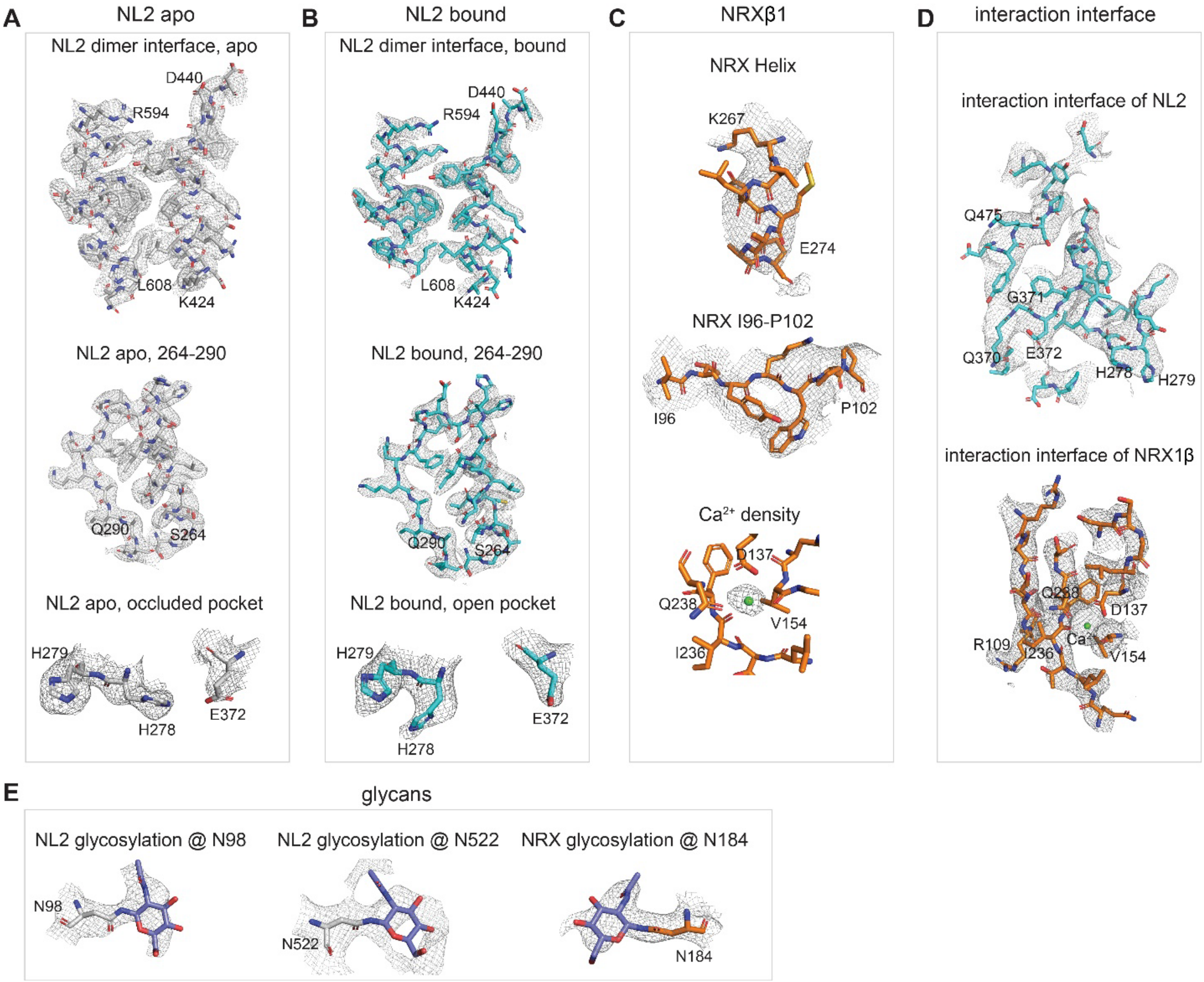

Figure S3. Density maps for representative regions. Regions of (**A**) NL2 apo (gray), (**B**) NL2 bound (cyan), (**C**) NRXβ1 (orange), (**D**) NL2- NRXβ1 interaction interface and (**E**) N-linked glycans (light blue) are shown.

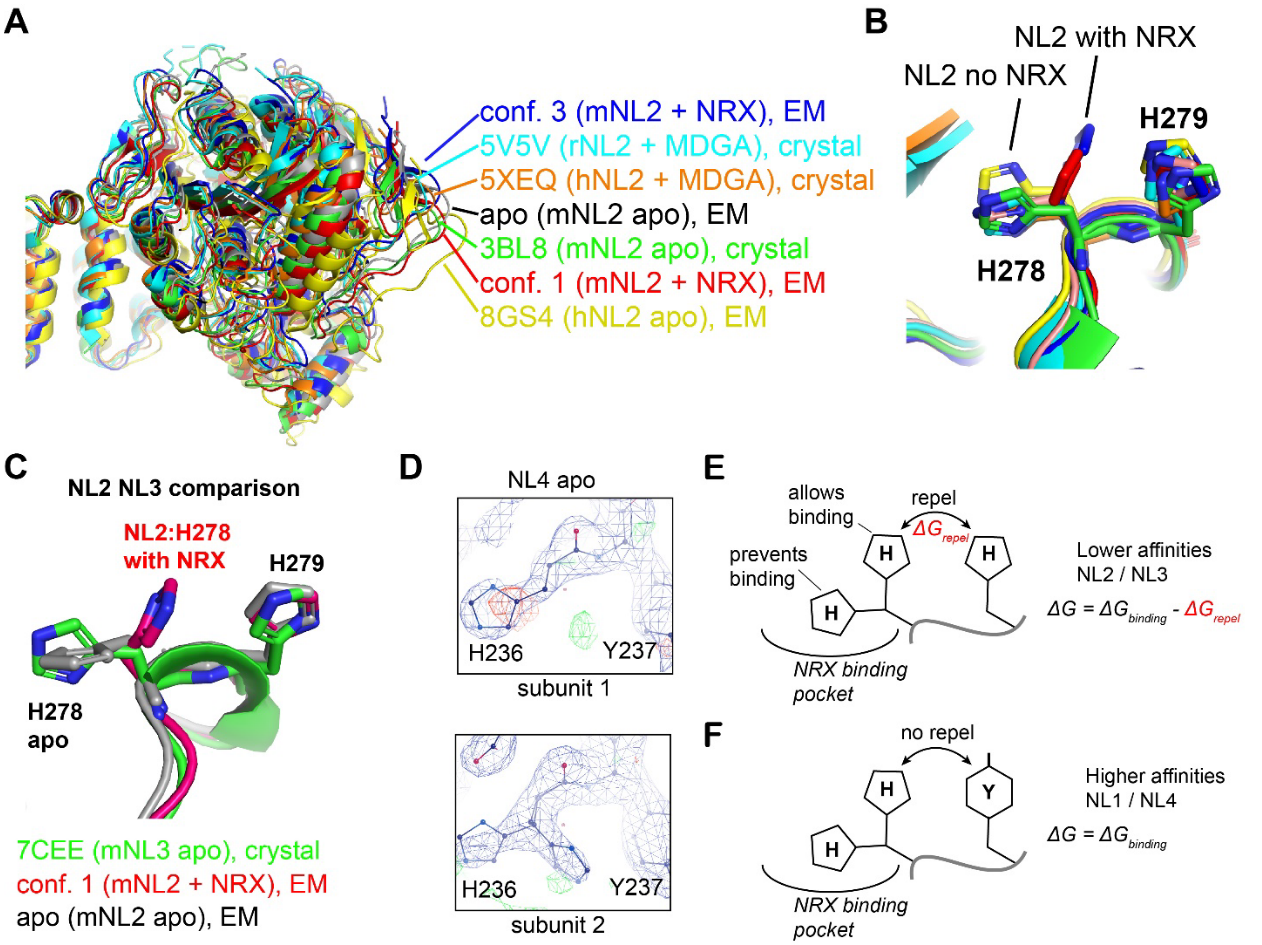

Figure S4. Comparison of available neuroligin structures. (**A**) A comparison of one NL2 subunit orientation across reported NL2 structures, with the other subunit aligned. The structures resolved here: apo, conf. 1 and conf. 3 are overlaid as reference. PDB IDs are listed. (**B**) Stick representations of NL2:H278 and H279 in structures listed in (A). (**C**) Stick representations of H278 and H279 in NL2 apo (gray), NL2-NRXβ1 (red, conf. 1), and NL3 apo (green). (**D**) NL4 apo structure (PDB ID: 3BE8) near H236 and Y237, with $2F_o$-F_c (1.5 rmsd, blue), F_o-F_c (2.6 rmsd, red: negative, green: positive) maps shown for subunit 1 (upper) and 2 (lower). Model of how (**E**) HH and (**F**) HY sequences contribute to lower or higher binding affinities, respectively.

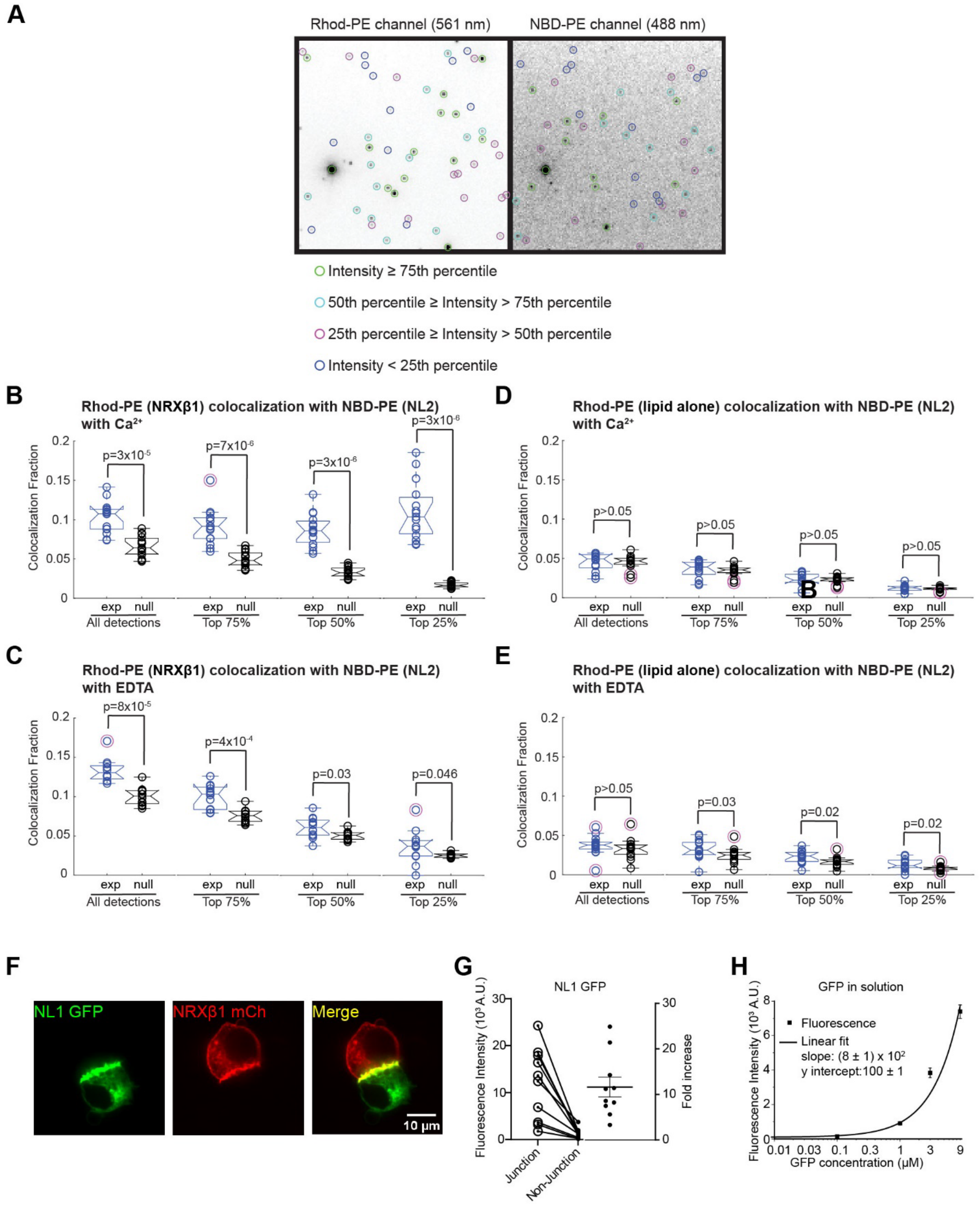

Figure S5. Object-based colocalization analysis. (**A**) Illustration of detections with object intensities falling into indicated ranges. (**B** and **C**) Object-based co-localization analysis of NRXβ1

(Rhod-PE) with NL2 (NBD-PE) vesicles in the presence of (**B**) Ca^{2+} and (**C**) EDTA. (**D** and **E**) Co-localization analysis of lipid alone (Rhod-PE) with NL2 (NBD-PE) vesicles in the presence of (**D**) Ca^{2+} and (**E**) EDTA. In B-E, for data presentation description and number of data points used in analysis, see Fig. 5. (**F**) Representative confocal micrographs of co-cultured cells expressing NRXβ1-mCherry (red) and NL1-GFP (Green). (**G**) Quantification of NL2-GFP intensities within and outside of cellular junctions. (**H**) Calibration of fluorescence intensity against GFP in solution.

Supplementary Table 1 Cryo-EM data collection, refinement and validation statistics

	NL2 Dimer	NL2-NRXβ1 (conf. 0)	NL2-NRXβ1 (conf. 1)	NL2-NRXβ1 (conf. 2)	NL2-NRXβ1 (conf. 3)
**PDB ID**	8G7D	8G7Y	8G7Z	8G81	8G80
**Data collection and processing**					
Magnification	105,000	105,000	105,000	105,000	105,000
Voltage (kV)	300	300	300	300	300
Electron exposure (e–/Å^2)	90	70	70	70	70
Defocus range (μm)	-1.0 to -2.5	-1.0 to -2.5	-1.0 to -2.5	-1.0 to -2.5	-1.0 to -2.5
Pixel size (Å)	0.83	0.83	0.83	0.83	0.83
Symmetry imposed	*C1*	*C1*	*C1*	*C1*	*C1*
Initial particles(no.)	674,761	2,964,465	2,964,465	2,964,465	2,964,465
Final particles (no.)	77,577	46,435	51,041	78,367	50,603
Map resolution (Å)	3.28	3.92	3.22	3.22	3.25
FSC threshold	0.143	0.143	0.143	0.143	0.143
**Refinement**					
Initial model used (PDB code)	3BL8	3BIW	3BIW	3BIW	3BIW
Model resolution (Å)	3.28	3.92	3.22	3.22	3.25
FSC threshold	0.5	0.5	0.5	0.5	0.5
Model composition					
Non-hydrogen atoms	8346	19146	11094	11148	11067
Protein residues	1076	1258	1427	1432	1425
Ligands	NAG:4	NAG:5 CA:1	NAG:6 CA:2	NAG:6 CA:2	NAG:6 CA:2
B factors (Å^2)					
Protein	77.52	157.02	88.21	120.42	74.30
Ligand	136.32	186.97	128.38	166.43	142.03
R.m.s. deviations					
Bond lengths (Å)	0.003	0.003	0.004	0.003	0.002
Bond angles (°)	0.516	0.542	0.582	0.563	0.502
Validation					
MolProbity score	1.77	1.98	1.90	1.76	1.78
Clashscore	6.07	10.60	8.99	8.31	8.55
Rotamer outliers (%)	0	0	0	0	0
Ramachandran plot					
Favored (%)	93.33	93.32	93.62	95.55	95.39
Allowed (%)	6.67	6.60	6.38	4.45	4.61
Outliers (%)	0.0	0.0	0.0	0.0	0.0
